# 

**Published:** 1897-01-09

**Authors:** 


ABSOLUTELY PURE, therefore BEST,

CADBURY'S COCOA

"The standard of highest purity at present attainable."—*Lancet.*

CADBURY'S COCOA

NO ALKALIES USED.

### THE HOSPITAL

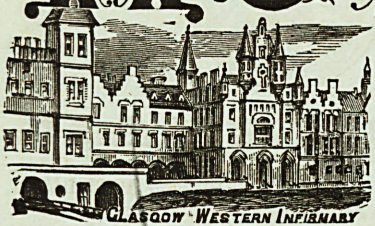

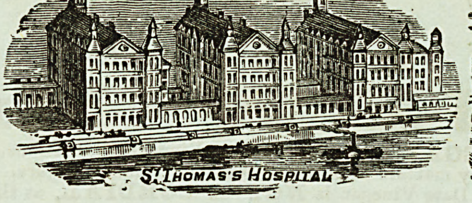

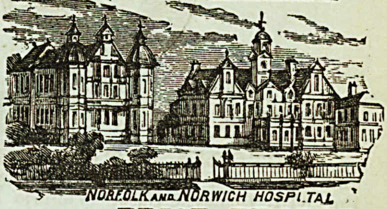

No. 537. Vol. XXI.

SATURDAY, JAN. 9TH, 1897.

[Registered at the General Post Office as a Newspaper for transmission in the United Kingdom and Abroad.]

PRICE TWOPENCE.

Per Annum, 10s. 6d., post free.

Monthly Parts, 6d.; post free, 8d.

Ditto per Annum, 8s. 6d., post free.

